# Intra‐arterial chemoradiotherapy for locally advanced buccal mucosal cancer in an elderly patient with multiple comorbidities: A case report

**DOI:** 10.1002/cnr2.1629

**Published:** 2022-05-25

**Authors:** Akito Taniguchi, Yutaka Toyomasu, Akinori Takada, Takamitsu Mase, Kazuto Kurohara, Kazuki Omori, Yui Nanpei, Tomoko Kawamura, Hajime Sakuma, Yoshihito Nomoto

**Affiliations:** ^1^ Department of Radiology Mie University Hospital Tsu Mie Japan; ^2^ Department of Oral and Maxillofacial Surgery Mie University Hospital Tsu Mie Japan

**Keywords:** buccal mucosal cancer, cisplatin, comorbidities, intra‐arterial chemoradiotherapy, squamous cell carcinoma

## Abstract

**Background:**

The management of locally advanced oral cavity squamous cell carcinoma (LA‐OCScc) in elderly patients with multiple comorbidities is difficult.

**Case:**

We report the case of an elderly patient with buccal mucosal squamous cell carcinoma as well as chronic renal dysfunction, hepatic cirrhosis, rheumatoid arthritis, and metachronous diffuse large B‐cell lymphoma. We performed radiation therapy (a total dose of 70 Gy in 35 fractions) and six cycles of intra‐arterial chemotherapy with 40 mg/m^2^ cisplatin per week.

After treatment, the tumor completely disappeared, and there was no recurrence or deterioration of comorbidities during the 12‐month follow‐up period.

**Conclusion:**

Intra‐arterial chemoradiotherapy may be a good treatment option for LA‐OCScc in elderly patients with multiple comorbidities.

## 1 INTRODUCTION

Head and neck cancer is a common malignancy that causes more than 300 000 deaths per year worldwide.^1^ Approximately 25% of patients with head and neck cancer are over the age of 70 years.^2^, ^3^ Various prognostic factors of head and neck cancer have been reported. Several factors, such as age, comorbidity, and human papilloma virus status, often have an influence on initial treatment strategy.^4^, ^5^, ^6^

Surgery followed by postoperative chemoradiotherapy is the standard treatment for locally advanced oral cavity squamous cell carcinoma (LA‐OCScc). Up‐front surgery for LA‐OCScc has been shown to provide a substantial survival benefit compared to definitive radiotherapy with or without chemotherapy in a small randomized trial^7^ and several retrospective studies.^8^, ^9^, ^10^ However, the number of patients with LA‐OCScc receiving non‐surgical treatment has been increasing.^5^ In particular, patients aged 75 years or older have received non‐surgical treatment more often than younger patients.^10^

Concurrent chemoradiotherapy (CRT) is the most acceptable treatment approach for the non‐surgical treatment of LA‐OCScc. The standard treatment regimen consists of three cycles of cisplatin (100 mg/m^2^) administered on days 1, 22, and 43 during radiotherapy (70 Gy in 35 fractions).^11^, ^12^ Since CRT improves survival outcomes achieved after radiotherapy alone, in addition to an increase in side effects, the management of toxicities has been important.^13^, ^14^ Several studies have shown that elderly patients can achieve outcomes similar to those of younger patients who receive aggressive treatments.^15^, ^16^ Nevertheless, elderly patients often receive less aggressive treatments, such as radiotherapy alone, compared with younger patients with the same disease status.^17^ This tendency is remarkable in elderly patients with multiple comorbidities. In the elderly, complications such as renal dysfunction have a significant impact on life expectancy; therefore, it is important to make aggressive treatment choices if the patient can tolerate the treatment.

In a randomized controlled phase 3 trial in patients with locally advanced unresectable head and neck cancer, intra‐arterial chemoradiotherapy (IA‐CRT) was not superior to intravenous chemoradiotherapy (IV‐CRT) in relation to outcomes, but rates of local control and overall survival were similar between the two groups.^18^ Superselective intra‐arterial chemotherapy (IA‐CT) is divided into two types: selective arterial infusion through the femoral artery^19^, ^20^ or retrograde selective infusion via the superficial temporal artery (STA) and/or occipital artery (OA).^21^, ^22^, ^23^ The former method is standard, but some studies have reported the risk of neurologic toxicity.^18^, ^20^ In contrast, the conventional method of catheterization through the STA is rarely associated with neurologic toxicity, but results in inadequate selectivity in some patients in whom the tumor was supplied by multiple arteries. Our previous report described treating head and neck cancer with multiple tumor‐feeding arteries with retrograde superselective IA‐CT using an external carotid arterial sheath (ECAS) system.^23^ Superselective IA‐CT using the ECAS system can be performed in elderly patients with carotid artery stenosis. However, there is insufficient evidence to show the efficacy and safety of retrograde intra‐arterial chemotherapy with concurrent chemotherapy in elderly patients with multiple comorbidities. In this study, we report a case study using IA‐CT with concurrent radiotherapy in an elderly patient with LA‐OCScc as well as chronic renal dysfunction, hepatic cirrhosis, rheumatoid arthritis, and metachronous diffuse large B‐cell lymphoma.

## 2 CASE REPORT

**FIGURE 1:**
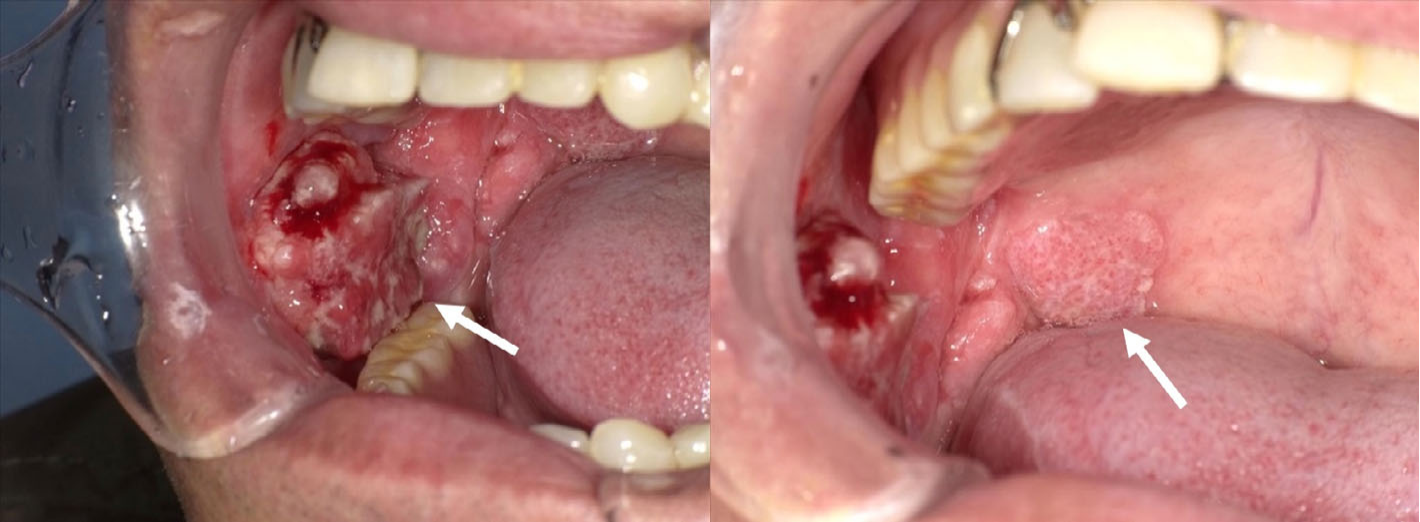
Photograph of the oral cavity showing a mass in right buccal mucosa (left) and a mucosal lesion on the soft palate (right).

**FIGURE 2:**
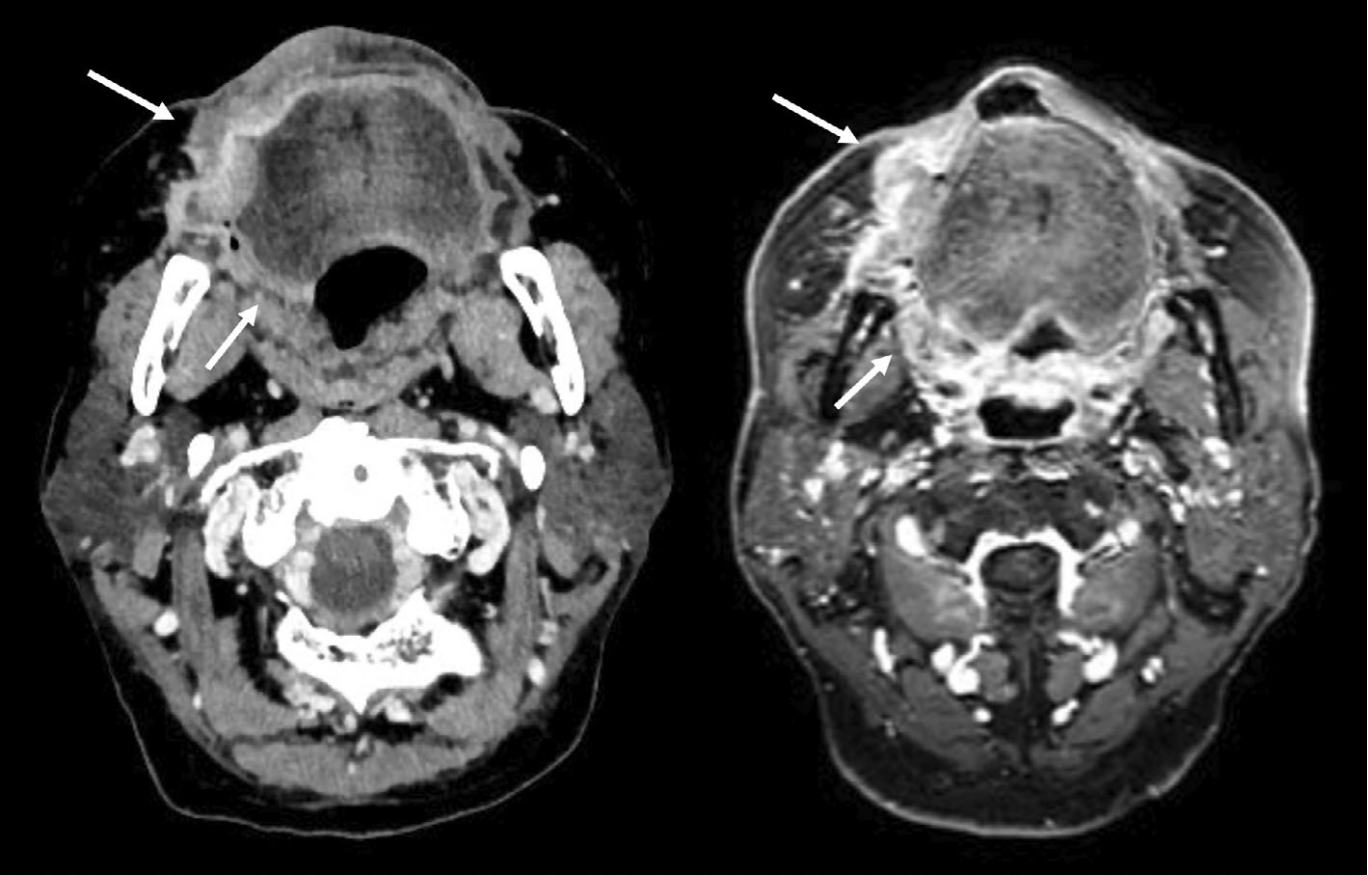
Computed tomography scan and T1‐weighted contrast‐enhanced magnetic resonance imaging scans. Axial views with contrast enhancement showing the tumor (tumor volume: 33 cm^3^).

A 76‐year‐old man was referred to Mie University Hospital due to exacerbation of swelling of the right buccal mucosa. He had a history of cirrhosis (Child‐Pugh class B) and chronic renal dysfunction. He was taking prednisolone for rheumatoid arthritis. In addition, he had maintained complete response for 3 years after treatment with six cycles of the rituximab, cyclophosphamide, doxorubicin, vincristine, and prednisone regimen for methotrexate‐related malignant lymphoma (DLBCL). Physical examination revealed a mass of the right buccal mucosa (28 × 25 mm) and a mucosal lesion on the soft palate (Figure 1). He had redness and induration on the skin near the right corner of the mouth. Computed tomography (CT) with contrast showed an enhancing tumor in the right buccal mucosa, and the lesion was suspected to extend to the soft palate (Figure 2). Magnetic resonance imaging (MRI) revealed similar findings. An enhancing effect was observed on the skin near the right corner of the mouth, and skin infiltration was suspected. No infiltration of the mandible was observed. There were no findings suggestive of metastatic lymph nodes or distant metastases. Imaging studies and buccal mucosal biopsy indicated a buccal mucosal squamous cell carcinoma (cT4aN0M0, stage IVA).

**FIGURE 3:**
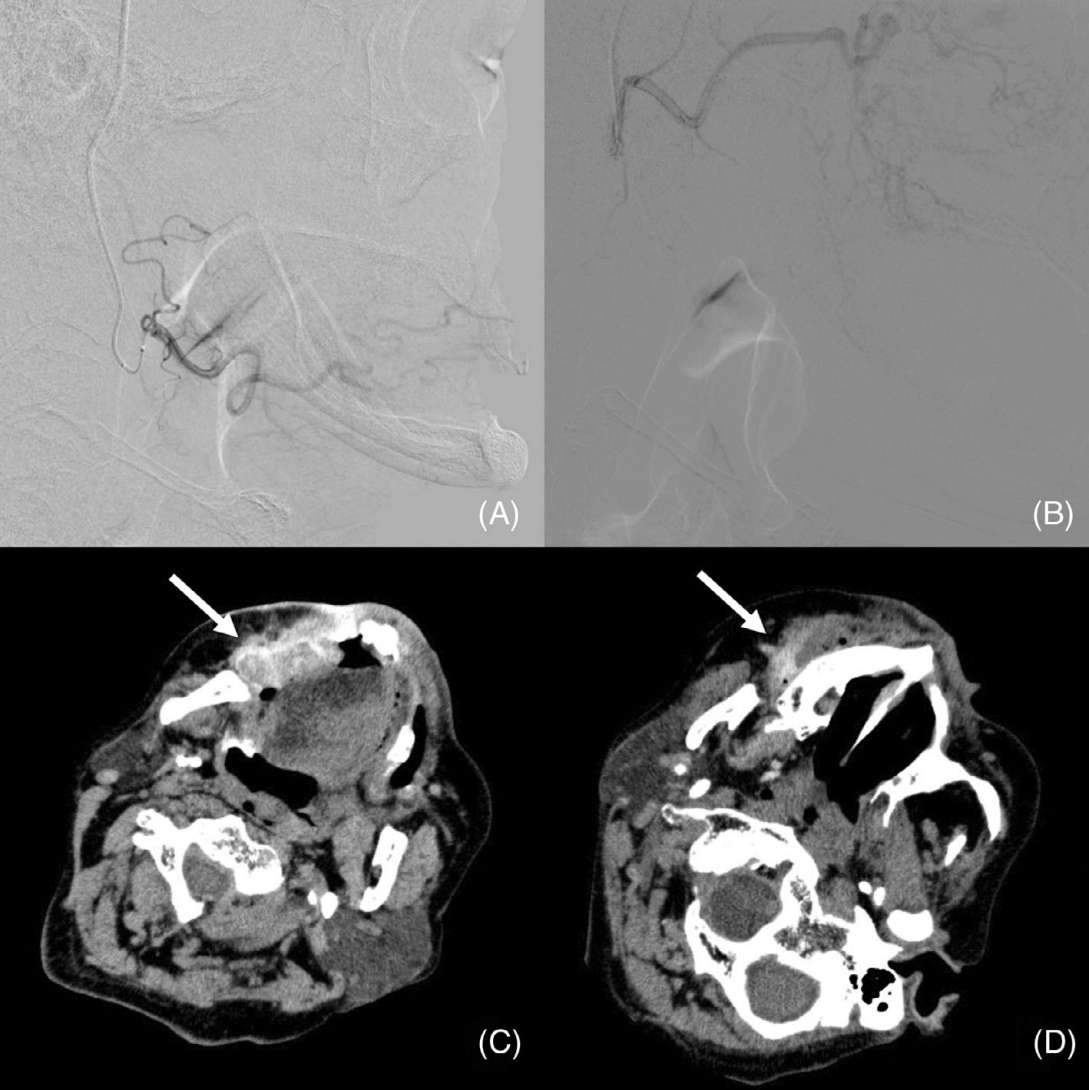
Digital subtraction angiograms (DSAs) and axial views of angio‐computed tomography (CT) images through retrograde intra‐arterial infusion. The catheters are selectively inserted into the right facial artery (FA) (A) and into the right maxillary artery (MA) (B). Axial view of the angio‐CT images after infusion of contrast medium through the catheters. Almost the whole area of the tumor is stained through the right FA (C). The upper margin of the tumor is stained through the right MA (D).

If surgery were to be performed, it would have required extensive resection and subsequent reconstructive surgery, not just the extent of the tumor invasion. Problems such as plastic surgery, anesthetics, and postoperative functional impairment were also considered. Therefore, the patient requested IA‐CRT. Sequential boost intensity‐modulated radiotherapy was delivered at a total dose of 70 Gy. The gross tumor volume (GTV) included the primary tumor (GTVp) and clinically metastatic lymph nodes (GTVn). Although there were no lymph node metastases at the time of staging, GTVn was defined as lymph nodes that showed shrinkage as irradiation proceeded. The clinical target volume 1 (CTV1) included the GTVp plus a 5‐ to 10‐mm margin and elective nodal regions (ipsilateral levels I to V and the contralateral levels I to IV). CTV2 included GTV plus a 5‐ to 10 mm margin. CTV3 included GTVn plus a 5‐mm margin. Three planning target volumes (PTV1‐3) were created that encompassed the corresponding CTV with a setup margin of 3 mm. Dose prescriptions were 40 Gy in 20 fractions for PTV1, 60 Gy in 30 fractions for PTV2 and 70 Gy in 35 fractions for PTV3. A catheter was inserted through the right superficial temporal artery and placed between the right facial and maxillary arteries (Figure 3). CT angiography and the arterial infusion of indigo carmine were performed to detect tumor‐feeding arteries. His creatinine clearance was 45.5 ml/min and we planned to administer cisplatin 40 mg/m^2^/week. Sodium thiosulfate (STS; 16 g/m^2^) was used for neutralization. Cisplatin 65 mg was injected into the right facial artery (35–40 mg) and the right maxillary artery (25–30 mg). After the completion of the three courses, neutropenia grade 3 (Common Terminology Criteria for Adverse Events, version 5.0) was observed and the treatment was temporarily discontinued. After confirming the recovery of neutrophils, chemotherapy was resumed and six courses were administered. The overall treatment time was 65 days.

**FIGURE 4:**
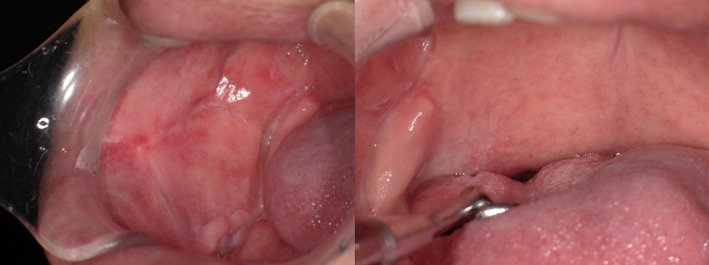
No recurrence or complications from treatment were observed at the 12‐month follow‐up.

Regarding acute adverse events, neutropenia was the only adverse event with grade 3 or higher. No exacerbation of liver or renal function was observed during the course of treatment or after the end of treatment. After the end of treatment, the tumor shrank significantly, and visual inspection and imaging showed a complete response (Figure 4). There were no symptoms of recurrent tumor or unexpected complications from treatment at the 12‐month follow‐up after the completion of intra‐arterial chemoradiotherapy.

## 3 DISCUSSION

Although the treatment of an elderly patient with head and neck tumor with multiple comorbidities is challenging, IA‐CRT is one of the curative treatments for inoperable patients. Here, we applied IA‐CRT to a patient with LA‐OCScc who was difficult to treat curatively due to age and basic diseases such as chronic renal failure and hepatic cirrhosis.

The standard treatment for LA‐OCScc is surgery and postoperative radiotherapy with or without chemotherapy. According to previous reports on treatment outcomes of surgery with postoperative CRT in elderly patients with head and neck cancer, the 3‐year overall survival (OS) and local control (LC) rates were 64% and 79%, respectively.^24^ However, major surgery is less frequently offered to elderly patients because the incidence of medical complications is significantly increased due to the presence of degenerative conditions and comorbidities.^25^, ^26^ With respect to the postoperative quality of life in elderly patients with locally advanced head and neck cancer undergoing major surgery, Khafif et al. reported that oral function was decreased by surgical intervention more severely in the elderly patient group than in the younger group.^26^

Concurrent CRT is the most acceptable nonsurgical treatment approach for LA‐OCScc. In a meta‐analysis of chemotherapy, Pignon et al. demonstrated an absolute OS improvement of 6.5% at 5 years after the addition of concurrent chemotherapy to radiotherapy.^12^ In a subgroup analysis, they noted a decreasing survival benefit of chemotherapy with age, specifically in patients who were 71 years old or older. By contrast, some studies showed similar outcomes with the same treatment between elderly and younger patients.^15^, ^16^ Two retrospective studies that investigated large national databases reported conflicting conclusions regarding the benefit of the addition of chemotherapy to radiotherapy on survival in elderly patients. However, both studies suggested that comorbidities are an important prognostic factor.^4^, ^27^ The Charlson comorbidity index (CCI) is a method of categorizing patients' comorbidities. Other studies including patients with head and neck squamous cell carcinoma treated with curative intended radiotherapy found that an increased level of comorbidity increased the risk of death.^28^ In this case, the CCI score was 7. The management of elderly patients with high CCI is controversial.^29^

Superselective IA‐CT for LA‐OCScc has the advantage of delivering a high concentration of chemotherapeutic agents to the tumor bed with fewer systemic toxic effects than systemic chemotherapy. Robbins et al. reported the use of intra‐arterial infusion of high‐dose cisplatin (150 mg/m^2^, weekly for 4 weeks) combined with STS for systemic cisplatin neutralization^19^, ^30^ and a high response rate of IA‐CRT. A randomized controlled phase 3 trial in patients with locally advanced unresectable head and neck cancer showed that IA‐CRT was not superior to IV‐CRT in terms of locoregional control and survival. However, outcomes in the IA‐CRT group were similar to those in the IV‐CRT group, and in a subgroup analysis, there was a significantly higher local and locoregional control rate in the IA‐CRT group for large (>30 ml) lateralized tumors. Some retrospective studies of IA‐CRT reported good outcomes for LA‐OCScc (Table 1). In a study of 134 patients treated with retrograde IA‐CRT with weekly carboplatin or cisplatin, the 3‐year LC and OS rates were 69% and 54%, respectively.^21^ Mitsudo et al. evaluated the treatment outcomes of 112 patients with stage III and IV OCScc treated with retrograde IA‐CRT with daily cisplatin and weekly docetaxel and reported 5‐year LC and OS rates of 79% and 71%, respectively.^22^ With respect to elderly patients with OCScc, Hayashi et al. evaluated the treatment outcomes of retrograde IA‐CRT with daily cisplatin and weekly docetaxel in 31 patients and reported 3‐year locoregional control and OS rates of 81% and 78%, respectively.^31^

**TABLE 1:** Local control and survival rates in patients with oral cancer treated with intra‐arterial chemoradiotherapy in previous studies

Author	Number of cases	Oral cancer rate (%)	Age (range)	Local control rate	Overall survival rate
Fuwa et al.^21^	134	100	Median 67 (25–89)	69%, 3 years	54%, 3 years
Mitsudo et al.^22^	112	100	Median 59 (28–87)	79%, 5 years	71%, 5 years
Hayashi et al.^31^	31	100	Median 83 (80–88)	81%, 3 years	78%, 3 years
Rasch et al.^18^	118	17	Mean 55	76%, 3 years	51%, 3 years

In addition to being elderly, our patient had moderate renal dysfunction, liver cirrhosis, and a history of chemotherapy. A reduction in the dosage of cisplatin is recommended in the presence of renal dysfunction, and the risk of bone marrow toxicity was anticipated to be higher due to cirrhosis and previous chemotherapy. When using cisplatin, it has been reported that the risk of neutropenia is lower with doses of 30–40 mg/m^2^ weekly compared to 100 mg/m^2^ tri weekly.^32^, ^33^ Additionally, the risk of exacerbating renal dysfunction can be reduced with STS. In this case, the patient was able to receive six cycles of IA‐CRT at 40 mg/m^2^ with STS. In regard to myelosuppression, grade 3 neutropenia was observed during the treatment period, whereas after treatment, neutrophils, hemoglobin, and platelets recovered to pre‐treatment levels. The outcome of this treatment was that the tumor was controlled for 12 months after the completion of IA‐CRT without impairing the patient's quality of life, such as renal dysfunction and dysphagia.

In conclusion, radiotherapy combined with intra‐arterial chemotherapy may be curative non‐surgical treatment option for locally advanced oral cancer in elderly patients with multiple comorbidities.

## AUTHOR CONTRIBUTIONS

**Akito Taniguchi:** Conceptualization (equal); writing – original draft (equal). **Yutaka Toyomasu:** Writing – original draft (equal). **Akinori Takada:** Writing – review and editing (equal). **Takamitsu Mase:** Resources (equal). **Kazuto Kurohara:** Resources (equal). **Kazuki Omori:** Resources (equal). **Yui Nanpei:** Investigation (equal). **Tomoko Kawamura:** Investigation (equal). **Hajime Sakuma:** Supervision (equal); writing – review and editing (equal). **Yoshihito Nomoto:** Supervision (equal); writing – review and editing (equal).

## CONFLICT OF INTEREST

The authors declare that they have no conflict of interest related to this study.

## ETHICS STATEMENT

This case report does not require institutional review board approval, as it only includes one patient.

## Data Availability

The data that support the findings of this study are available from the corresponding author upon reasonable request.

## References

[1] Sung H , Ferlay J , Siegel RL , et al. Global cancer statistics 2020: GLOBOCAN estimates of incidence and mortality worldwide for 36 cancers in 185 countries. CA Cancer J Clin. 2021;71:209‐249. 33538338 10.3322/caac.21660

[2] Muir CS , Fraumeni JF Jr , Doll R . The interpretation of time trends. Cancer Surv. 1994;19‐20:5‐21. 7895222

[3] Grenman R , Chevalier D , Gregoire V , Myers E , Rogers S . Treatment of head and neck cancer in the elderly: European consensus (panel 6) at the EUFOS congress in Vienna 2007. Eur Arch Otorhinolaryngol. 2010;267:1619‐1621. 20454970 10.1007/s00405-010-1263-6

[4] Amini A , Jones BL , McDermott JD , et al. Survival outcomes with concurrent chemoradiation for elderly patients with locally advanced head and neck cancer according to the National Cancer Data Base. Cancer. 2016;122:1533‐1543. 26969811 10.1002/cncr.29956

[5] Cannon RB , Sowder JC , Buchmann LO , et al. Increasing use of nonsurgical therapy in advanced‐stage oral cavity cancer: a population‐based study. Head Neck. 2017;39:82‐91. 27641220 10.1002/hed.24542

[6] Orlandi E , Iacovelli NA , Tombolini V , et al. Potential role of microbiome in oncogenesis, outcome prediction and therapeutic targeting for head and neck cancer. Oral Oncol. 2019;99:104453. 31683170 10.1016/j.oraloncology.2019.104453

[7] Iyer NG , Tan DS , Tan VK , et al. Randomized trial comparing surgery and adjuvant radiotherapy versus concurrent chemoradiotherapy in patients with advanced, nonmetastatic squamous cell carcinoma of the head and neck: 10‐year update and subset analysis. Cancer. 2015;121:1599‐1607. 25639864 10.1002/cncr.29251

[8] Murthy V , Agarwal JP , Laskar SG , et al. Analysis of prognostic factors in 1180 patients with oral cavity primary cancer treated with definitive or adjuvant radiotherapy. J Cancer Res Ther. 2010;6:282‐289. 21119254 10.4103/0973-1482.73360

[9] Sher DJ , Thotakura V , Balboni TA , et al. Treatment of oral cavity squamous cell carcinoma with adjuvant or definitive intensity‐modulated radiation therapy. Int J Radiat Oncol Biol Phys. 2011;81:e215‐e222. 21531515 10.1016/j.ijrobp.2011.02.023

[10] Fujiwara RJT , Burtness B , Husain ZA , et al. Treatment guidelines and patterns of care in oral cavity squamous cell carcinoma: primary surgical resection vs. nonsurgical treatment. Oral Oncol. 2017;71:129‐137. 28688680 10.1016/j.oraloncology.2017.06.013

[11] Adelstein DJ , Li Y , Adams GL , et al. An intergroup phase III comparison of standard radiation therapy and two schedules of concurrent chemoradiotherapy in patients with unresectable squamous cell head and neck cancer. J Clin Oncol. 2003;21:92‐98. 12506176 10.1200/JCO.2003.01.008

[12] Pignon JP , le Maitre A , Maillard E , Bourhis J , Group M‐NC . Meta‐analysis of chemotherapy in head and neck cancer (MACH‐NC): an update on 93 randomised trials and 17,346 patients. Radiother Oncol. 2009;92:4‐14. 19446902 10.1016/j.radonc.2009.04.014

[13] De Felice F , de Vincentiis M , Luzzi V , et al. Late radiation‐associated dysphagia in head and neck cancer patients: evidence, research and management. Oral Oncol. 2018;77:125‐130. 29362118 10.1016/j.oraloncology.2017.12.021

[14] De Felice F , de Vincentiis M , Valentini V , et al. Follow‐up program in head and neck cancer. Crit Rev Oncol Hematol. 2017;113:151‐155. 28427504 10.1016/j.critrevonc.2017.03.012

[15] Moye VA , Chandramouleeswaran S , Zhao N , et al. Elderly patients with squamous cell carcinoma of the head and neck and the benefit of multimodality therapy. Oncologist. 2015;20:159‐165. 25582139 10.1634/theoncologist.2013-0325 PMC4319622

[16] Michal SA , Adelstein DJ , Rybicki LA , et al. Multi‐agent concurrent chemoradiotherapy for locally advanced head and neck squamous cell cancer in the elderly. Head Neck. 2012;34:1147‐1152. 22021098 10.1002/hed.21891

[17] Juarez JE , Choi J , St John M , Abemayor E , TenNapel M , Chen AM . Patterns of care for elderly patients with locally advanced head and neck cancer. Int J Radiat Oncol Biol Phys. 2017;98:767‐774. 28366573 10.1016/j.ijrobp.2017.01.209

[18] Rasch CR , Hauptmann M , Schornagel J , et al. Intra‐arterial versus intravenous chemoradiation for advanced head and neck cancer: results of a randomized phase 3 trial. Cancer. 2010;116:2159‐2165. 20187094 10.1002/cncr.24916

[19] Robbins KT , Storniolo AM , Kerber C , Seagren S , Berson A , Howell SB . Rapid superselective high‐dose cisplatin infusion for advanced head and neck malignancies. Head Neck. 1992;14:364‐371. 1399569 10.1002/hed.2880140505

[20] Robbins KT , Kumar P , Harris J , et al. Supradose intra‐arterial cisplatin and concurrent radiation therapy for the treatment of stage IV head and neck squamous cell carcinoma is feasible and efficacious in a multi‐institutional setting: results of radiation therapy oncology group trial 9615. J Clin Oncol. 2005;23:1447‐1454. 15735120 10.1200/JCO.2005.03.168

[21] Fuwa N , Kodaira T , Furutani K , et al. Intra‐arterial chemoradiotherapy for locally advanced oral cavity cancer: analysis of therapeutic results in 134 cases. Br J Cancer. 2008;98:1039‐1045. 18283309 10.1038/sj.bjc.6604272 PMC2275486

[22] Mitsudo K , Koizumi T , Iida M , et al. Retrograde superselective intra‐arterial chemotherapy and daily concurrent radiotherapy for stage III and IV oral cancer: analysis of therapeutic results in 112 cases. Radiother Oncol. 2014;111:306‐310. 24746571 10.1016/j.radonc.2014.03.005

[23] Ii N , Fuwa N , Toyomasu Y , et al. A novel external carotid arterial sheath system for intra‐arterial infusion chemotherapy of head and neck cancer. Cardiovasc Intervent Radiol. 2017;40:1099‐1104. 28357576 10.1007/s00270-017-1635-z

[24] Airoldi M , Cortesina G , Giordano C , et al. Postoperative adjuvant chemoradiotherapy in older patients with head and neck cancer. Arch Otolaryngol Head Neck Surg. 2004;130:161‐166. 14967744 10.1001/archotol.130.2.161

[25] Blackwell KE , Azizzadeh B , Ayala C , Rawnsley JD . Octogenarian free flap reconstruction: complications and cost of therapy. Otolaryngol Head Neck Surg. 2002;126:301‐306. 11956539 10.1067/mhn.2002.122704

[26] Khafif A , Posen J , Yagil Y , et al. Quality of life in patients older than 75 years following major head and neck surgery. Head Neck. 2007;29:932‐939. 17615568 10.1002/hed.20635

[27] VanderWalde NA , Meyer AM , Deal AM , et al. Effectiveness of chemoradiation for head and neck cancer in an older patient population. Int J Radiat Oncol Biol Phys. 2014;89:30‐37. 24725687 10.1016/j.ijrobp.2014.01.053

[28] Boje CR , Dalton SO , Primdahl H , et al. Evaluation of comorbidity in 9388 head and neck cancer patients: a national cohort study from the DAHANCA database. Radiother Oncol. 2014;110:91‐97. 24412015 10.1016/j.radonc.2013.11.009

[29] Porceddu SV , Haddad RI . Management of elderly patients with locoregionally confined head and neck cancer. Lancet Oncol. 2017;18:e274‐e283. 28456589 10.1016/S1470-2045(17)30229-2

[30] Robbins KT , Storniolo AM , Kerber C , et al. Phase I study of highly selective supradose cisplatin infusions for advanced head and neck cancer. J Clin Oncol. 1994;12:2113‐2120. 7931481 10.1200/JCO.1994.12.10.2113

[31] Hayashi Y , Mitsudo K , Sakuma K , et al. Clinical outcomes of retrograde intra‐arterial chemotherapy concurrent with radiotherapy for elderly oral squamous cell carcinoma patients aged over 80 years old. Radiat Oncol. 2017;12:112. 28673362 10.1186/s13014-017-0847-3 PMC5496408

[32] Noronha V , Joshi A , Patil VM , et al. Once‐a‐week versus once‐every‐3‐weeks cisplatin chemoradiation for locally advanced head and neck cancer: a phase III randomized noninferiority trial. J Clin Oncol. 2018;36:1064‐1072. 29220295 10.1200/JCO.2017.74.9457

[33] Szturz P , Wouters K , Kiyota N , et al. Weekly low‐dose versus three‐weekly high‐dose cisplatin for concurrent chemoradiation in locoregionally advanced non‐nasopharyngeal head and neck cancer: a systematic review and meta‐analysis of aggregate data. Oncologist. 2017;22:1056‐1066. 28533474 10.1634/theoncologist.2017-0015 PMC5599190

